# Castration Instead of Lynching

**Published:** 1907-03

**Authors:** 


### SELECTIONS AND ABSTRACTS.

### CASTRATION INSTEAD OF LYNCHING.

When lynching overrides the law it is a stain upon the fair name of any State in which it occurs; it is not only a disgrace to the community, but it also establishes a precedent. In several of the States lynching has been used as a remedy in cases of criminal assault upon women. A revolting crime of this character should meet with severe punishment, but to violate law by lynching makes an additional crime in which a large number of people participate. The sober judgment of every one, it seems, should be to uphold the law, and it is difficult to see how good citizens can do otherwise. But suppose that lynching is considered justifiable, if it does not prevent crime, but on the contrary encourages it, it is worse than a failure. That it has failed there seems to be no doubt. The epidemic of criminal assault and lynching throughout the South has been made the text for an editorial in The Atlanta Journal-Record of Medicine. The editor says that the proper remedy is not being utilized in punishing the rapist, instead of having a deterrent effect it makes matters worse. The vicious class who commit these assaults seem to be under the impression that it is not a criminal who meets death but a martyr, and the same character of crime is again committed within a week after a lynching. The Journal-Record further says that in some instances the crime is committed with such brutality that nothing short of immediate death seems sufficient punishment. If, however, by a lynching we increase the danger of other women, should we not hesitate and devise, if possible, a remedy which, instead of making a temporary hero of the negro, will make of him a living example of the punishment to be meted out to all who thus attempt to gratify their bestial lust?

So intense is the animal passion of the negro that to be deprived of his sexual power would of necessity make him an object of ridicule and contempt among them.

In animal life we see clearly in the tractable gelding, the castrated mule, the ox, the capon, and in fact, in all animals and fowls, the effect of castration. In man, except in very rare instances, sexual power is lost by the removal of the testicles, and along with it he becomes docile, quiet and inoffensive. After castration his disgrace would be lasting in its moral effect.

To persuade an incensed mob that such punishment is preferable to lynching would be difficult until they could be shown the effect of emasculation, and that the negro would rather be hanged than to be deprived of sexual power so dear to him.

The kuklux klan, recently agitated, could be very useful in taking charge of these affairs in a regular way, and in assuring the frenzied friends that a punishment worse than death will be inflicted. The identity of the criminal could be ascertained by an orderly trial by the klan, and in this manner avoid punishing an innocent negro, as is often done in lynchings. The trial could be made more or less mysterious and impressive, for the negro stands in great awe of mysteries. This was clearly shown in Atlanta last winter by the belief among the negroes that there was a black wagon driven through the streets at night to capture subjects for the dissecting-rooms of the medical colleges. Absurd as is the idea, it made a profound impression upon the negroes and even those more or less intelligent were afraid to venture out on the darker streets at night.

An impressive trial by a ghost-like kuklux klan and a "ghost" physician or surgeon to perform the operation would make of it an event the "patient" would never forget, or cease to talk about and enlarge upon. This would do away with the martyrdom effect of lynching as well as the demoralizing results of mob law. The badge of disgrace and emasculation might be branded upon the face or forehead, as a warning, in the form of an "R" emblematic of the crime for which this punishment was and will be inflicted.

As long as many of the negroes believe, regardless of the revolting character of the crime, that lynchings are the result of race prejudice, this mode of punishment will only incense them and aggravate the condition. Certain of them think that their cause will be strengthened by these (according to them) martyrs, just as the Christian church spread more rapidly after the persecution by Nero.

All will admit that lynching is not only a failure, but that it is dangerous. Castration in no particular could be worse.

In the Virginia Medical Monthly for January, Ewell makes a plea for castration to prevent criminal assault, and says that lynchings have failed in their great object, namely, to prevent a repetition of the horrible act. He says that crime is on the increase and that in the South the number of criminal assaults upon women have within the last year been more than double what they were before in the same length of time. It is evident from the position taken by these gentlemen and others that lynching does not prevent the number of criminal assaults. hence there is every reason why men should obey the law, especially when the method adopted by them seems to increase rather than prevent crime. There should be a punishment to meet the crime, not only a punishment to the individual but one that will deter others from assaulting women. Thus far the problem has been unsolved and perhaps emasculation is a key to the situation. —Central States Medical Monitor, February, 1907.

